# A Preliminary Study of the Response of *Microcyclosporella mali* to Selected Essential Oils

**DOI:** 10.3390/molecules30153122

**Published:** 2025-07-25

**Authors:** Elżbieta Paduch-Cichal, Wojciech Wakuliński, Anna Wilkos, Katarzyna Bączek, Olga Kosakowska, Zenon Węglarz, Ewa Mirzwa-Mróz

**Affiliations:** 1 Department of Plant Protection, Institute of Horticultural Sciences, Warsaw University of Life Sciences-SGGW (WULS-SGGW), 166 Nowoursynowska Str., 02-787 Warsaw, Poland; 2 Department of Vegetable and Medicinal Plants, Institute of Horticultural Sciences, Warsaw University of Life Sciences-SGGW (WULS-SGGW), 166 Nowoursynowska Str., 02-787 Warsaw, Poland

**Keywords:** sooty blotch, essential oils, costmary, Greek oregano, thyme, flow cytometry, MIC, MDF

## Abstract

In Poland, the main causal agent of sooty blotch and flyspeck disease is the fungus *Microcyclosporella mali* J.Frank, Schroers et Crous, which is most commonly isolated from the spots found on apples. The aim of the paper was to study the effects of essential oils extracted from Greek oregano, thyme and costmary on *M. mali*. Analysis of the essential oils was conducted using gas chromatography–mass spectrometry (GC–MS) with a flame ionization detector (FID). The Greek oregano essential oil was classified to the carvacrol chemotype, while the thyme and costmary were classified to the thymol and the β-thujone chemotypes, respectively. The influence of these essential oils on the viability of the *M. mali* conidia was analysed cytometrically. The Greek oregano oil was characterised by the significantly highest activity against the *M. mali* spores. The regression analysis performed showed the occurrence of a significant linear relationship between the viability of the conidia and the concentration of the essential oils, which was then the basis for the determination of MICs and MFCs. The values of these parameters in the case of the Greek oregano oil were 0.9 and 0.4%, respectively, and for the thyme oil they were 1.2 and 2.4%.

## 1. Introduction

The apple tree is the basic species of fruit tree grown in the temperate climate zone, with apples being among the most frequently consumed fresh fruits and also being a valued raw material in the processing industry. The popularity of apples among consumers mainly results from their nutritional, health and dietary attributes. As such, a key issue is to improve the quality of apples in a way corresponding to the increasing requirements of consumers [1].

Sooty blotch and flyspeck disease causes disfigurement of fruits and is primarily found in orchards in the USA, Canada, Brazil, China, Germany, Slovenia, Serbia, Montenegro, Turkey, Norway, the Netherlands, England and Poland [2,3,4,5,6,7,8,9,10,11]. The aetiology of sooty blotch and flyspeck is complex. This disease is caused by several species of fungi belonging to the sooty blotch and flyspeck (SBFS) complex. In Poland, the complex consists of five fungal species, and the main causal agent of the disease is considered to be the species *Microcyclosporella mali* J.Frank, Schroers et Crous, which is most commonly isolated from the spots found on apples [12]. *M. mali* belongs to the anamorphic fungi of the kingdom Fungi, type Ascomycota, class Dothideomycetes, subclass Dothideomycetidae, order Mycosphaerellales (formerly Capnodiales), family Mycosphaerellaceae. The mycelial hyphae of this fungus are branched, light brown and from smooth to slightly papillary. The phialophores are reduced only to conidiogenous cells with dimensions of 5–7 × 1–2 μm, which are most frequently formed intrahyphally and sporadically at the ends of the hyphae. They are cylindrical to ampullate, smooth, mono- or polyblastic with flat bright scars of a size of 1–2 μm and form the conidia sympodially [7].

Although it does not directly decrease the yield of fruits, sooty blotch and flyspeck clearly worsens fruit quality. In the USA, even in commercial orchards with proper chemical protection, the losses resulting from the presence of this disease may range from 5 to 10%. As such, in some USA states, to prevent the occurrence of sooty blotch and flyspeck, it is necessary for additional chemical treatments to be carried out in the summer period [13].

In Poland, in commercial orchards with properly conducted chemical protection against apple scab (*Venturia inaequalis* (Cooke) G. Winter), there is virtually no occurrence of sooty blotch and flyspeck. However, the disease begins to pose a problem in plantings of scab-resistant varieties where there is no need to apply the full chemical protection against apple scab [14,15,16]. This disease is also increasingly recorded in organic orchards [12] in which, unlike the commercial orchards, the use of fungicides is very limited. Only sulfur- and copper-containing preparations are allowed, the use of which is characterised by many limitations and is less efficient compared to synthetic products [16]. Therefore, one of the more important factors determining the success of apple cultivation in an organic system, apart from the agrotechnical treatments, is the correct cutting of trees (enabling a faster drying rate of leaves and fruits) and thinning of buds [15] and selection of an appropriate variety (with resistance or little susceptibility to diseases and pests) [17].

Recently, there has been an increasing interest in plant-derived preparations, which are environmentally friendly and can be used as natural biopesticides to limit plant infestation. What appears to be promising are the results of research on the use of essential oils (EOs) extracted from nearly 18,000 oil-yielding plant species in the protection of plants against agrophages, especially as they are considered to be harmless to warm-blooded organisms and environmentally friendly. In modern plant protection, especially organic crop protection, they can be a good alternative to traditional plant protection products [18]. Therefore, the aim of the research presented in this paper was to study the response of the *M. mali* fungus to the effects of different concentrations of essential oils extracted from Greek oregano, thyme and costmary.

## 2. Results

### 2.1. EO Composition

In the Greek oregano essential oil (EO), 27 compounds were detected, making up 97.28% of the sample. Monoterpenes formed a fundamental part of this EO. Phenolic monoterpenes were present at the highest amount, with carvacrol being clearly dominant (60.65%) and with thymol only making up a small proportion (0.98%). Monoterpene hydrocarbons, which constituted 30.28%, were mainly represented by γ-terpinene (17.04%) and p-cymene (5.43%), while oxygenated monoterpenes (4.20%) included, among others, bornyl acetate, linalool, terpinen-4-ol and α-terpineol. The fraction of sesquiterpenes contained hydrocarbons (germacrene D) and oxygenated compounds such as (−)-spathulenol and α-bisabolol.

In the case of the thyme EO, 26 compounds were identified, comprising 97.35% of the total fraction. Phenolic monoterpenes were the dominant group in this EO (60.65%). Here, thymol amounted to 57.06%, while carvacrol accounted for 3.59%. Monoterpene hydrocarbons, with the highest proportion of p-cymene (16.56%) and γ-terpinene (7.33%), made up 29.31% of the sample. Oxygenated monoterpenes (3.72%) were mainly represented by linalool, terpinen-4-ol, borneol and bornyl acetate. The proportions of sesquiterpenes were at levels of 2.85% (sesquiterpene hydrocarbons) and 0.44% (oxygenated sesquiterpenes).

With regard to the costmary EO, 24 compounds were identified, accounting for 98.36% of the sample. Clear dominance was noted here for oxygenated monoterpenes (95.23%), mainly represented by thujones (β-thujone 89.38%; α-thujone 2.81%). The proportions of other fractions were found to be low, e.g., monoterpene hydrocarbons (1.99%), phenolic monoterpenes (0.51%), and sesquiterpenes (0.35%) (Table 1).

### 2.2. Flow Cytometry Analysis

**Figure 1:**
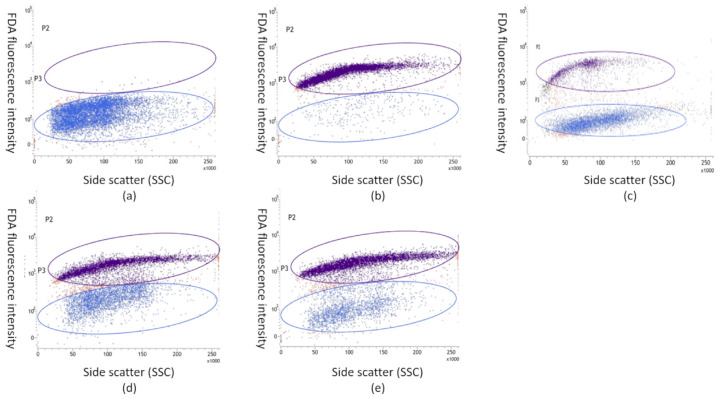
Dot plot of FDA versus integrity of the cell population assessed using side scatter (SSC) of *Microcyclosporella mali* spores. The blue dots represent non-viable conidia, while the purple coloration signifies viable ones. Population of spores (**a**) after being exposed to high temperature, (**b**) without treatment and (**c**) after incubation in Greek oregano (**d**), thyme and (**e**) costmary essential oil solutions.

**Figure 2:**
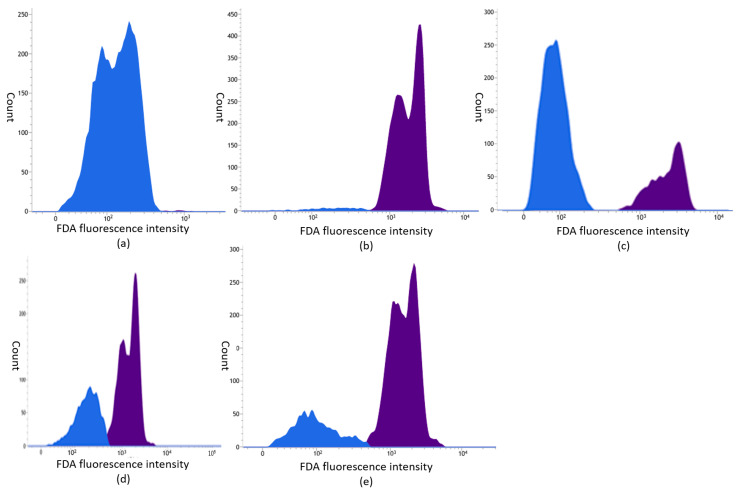
Histogram plot of *Microcyclosporella mali* conidia stained with FDA. The blue histograms represent non-viable conidia, while the purple ones represent viable ones. Population of spores (**a**) after being exposed to high temperature, (**b**) without treatment and (**c**) after incubation in Greek oregano, (**d**) thyme and (**e**) costmary essential oil solutions.

The initial step in the flow cytometric analysis involved separating debris from the *M. mali* spore (conidia) population using forward and side light scatter. Subsequently, viable *M. mali* spores were identified by gating subpopulations based on fluorescence: FDA-positive events (viable conidia) were distinguished from FDA-negative events (non-viable). The proportion of viable spores within the total spore population for each sample was presented in histograms (Figure 1a–e and Figure 2a–e), which served as the basis for quantitative analysis.

**Figure 3:**
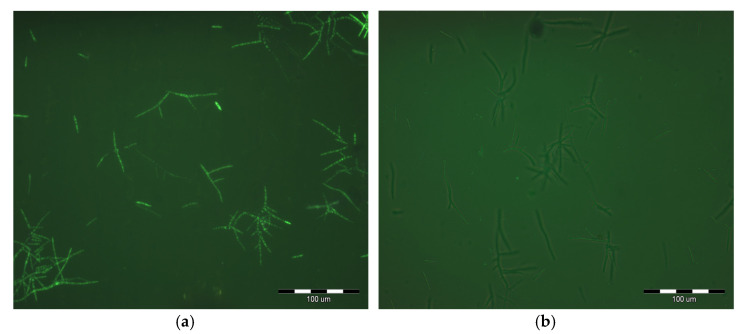
(**a**) *Microcyclosporella mali* conidia emitting green light (viable). (**b**) *Microcyclosporella mali* conidia not emitting green light (non-viable) (photo A. Wilkos).

The photograph displays the typical small, hyaline and multiseptate conidia of *M. mali*, measuring approximately 2.5 µm by 13–36 µm. After staining with fluorescein diacetate (FDA), both the cell wall and cytoplasm exhibit a distinct light-green fluorescence, characteristic of living cells (Figure 3a). This fluorescence arises only in metabolically active cells, where the non-fluorescent FDA is enzymatically converted into its fluorescent form. In contrast, spores previously exposed to high temperatures showed a complete absence of fluorescence (Figure 3b). Fluorescence microscopy clearly demonstrated the ability to differentiate between live and dead *M. mali* conidia using FDA staining. This differentiation is strongly influenced by the structural properties of the spores, particularly the absence of melanin in the cell wall, which otherwise could mask fluorescence.

### 2.3. Influence of the Essential Oils on the Viability of the M. mali Conidia

Based on the three-way analysis of variance performed (an essential oil × concentration of the essential oil × incubation periods), there was a significant influence for each of the above-mentioned factors and the interaction thereof on the viability of the *M. mali* conidia (Table S1).

The Newman–Keuls multiple comparisons test showed statistically significant differences in the influence of the three essential oils on the mortality of the conidia of the fungus. The oil obtained from Greek oregano turned out to be the most effective (Table 2). This oil was selected for studies on the viability of the conidia of the fungus after 15 min, 2 h and 24 h.

**Figure 4:**
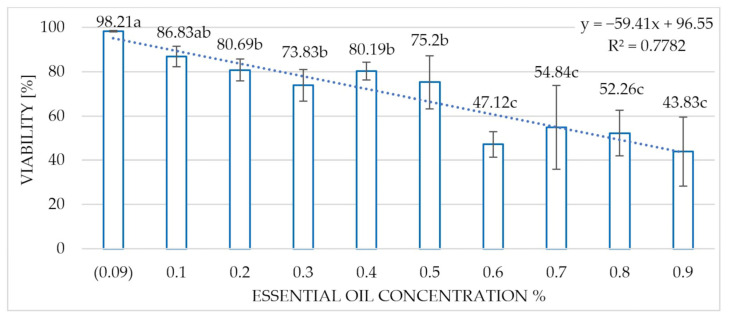
Influence of the concentration of Greek oregano essential oil on the viability of the *M. mali* conidia after 15 min of incubation. Homogeneous groups according to the Newman–Keuls test. Values marked with the same letter are not significantly different at the significance level of *p* = 0.05.

The concentration of the Greek oregano essential oil was determined to have a significant influence on the viability of the conidia of the fungus after only 15 min of incubation, and the most effective fungistatic effect was observed at oil concentrations from 0.7 to 0.9%, where the viability of the spores ranged from 54.84 to 43.83% (Figure 4).

**Figure 5:**
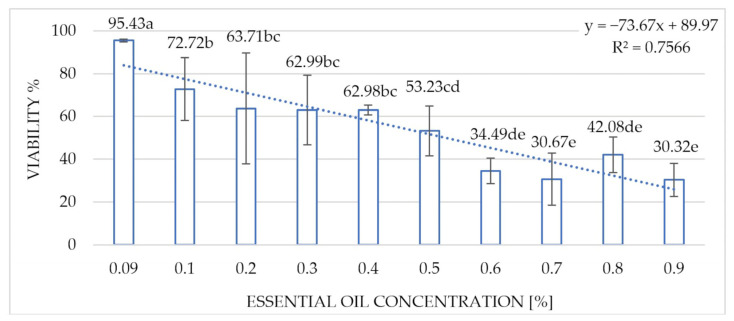
Influence of the concentration of the Greek oregano essential oil on the viability of the *Microcyclosporella mali* conidia after 2 h of incubation. Homogeneous groups according to the Newman–Keuls test. Values marked with the same letter are not significantly different at the significance level of *p* = 0.05.

The results of the studies on the viability of the *Microcyclosporella mali* conidia performed after 2 h of incubation showed statistically significant differences in the fungistatic effect of the essential oil against the fungus after applying the oil concentrations of 0.1–0.9%, with the viability of the conidia of the fungus ranging from 72.72 to 30.32% (Figure 5).

**Figure 6:**
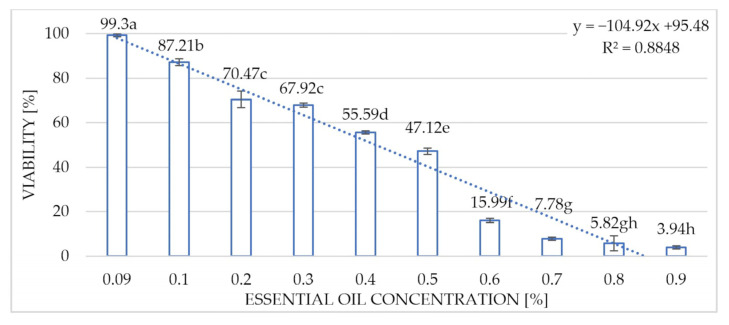
Influence of the concentration of the Greek oregano essential oil on the viability of the *Microcyclosporella mali* conidia after 24 h of incubation. Homogeneous groups according to the Newman–Keuls test. Values marked with the same letter are not significantly different at the significance level of *p* = 0.05.

The most efficient fungistatic effect of the Greek oregano oil against the *M. mali* conidia was shown to occur after 24 h of incubation. The statistically significant influence of the concentration applied was visible in terms of the viability of the conidia of the fungus. As the concentration of the essential oil was increased, a decrease in the viability of the conidia of the fungus occurred. At the lowest concentration (0.09%) of the Greek oregano EO used in the studies, the percentage of viable conidia was 99.3%, while the viability was only 3.94% when using the highest concentration of 0.9% (Figure 6).

### 2.4. Determination of the Minimum Inhibitory Concentration (MIC) and Minimum Fungicidal Concentration (MFC)

Based on regression analysis, linear regression equations were determined, showing the relationship between the applied concentration of an essential oil and the viability of the *M. mali* conidia. These equations were developed for the essential oils extracted from Greek oregano and thyme but not for the costmary oil, which was discarded in the first stage of the studies because its fungistatic effect against the conidia was very weak compared to the other two essential oils, even when applying high concentrations of the essential oil (Table 3).

The determined regression equations (Table S2) allowed us to determine the MIC and MFC values for the Greek oregano and thyme essential oils.

For the Greek oregano essential oil:

- The MIC values were 0.8%, 0.5% and 0.4% after 15 min, 2 h and 24 h, respectively;
- The MFC values were 1.6%, 1.3% and 0.9% after 15 min, 2 h and 24 h, respectively.

For the Greek oregano essential oil, the analysis of the obtained MIC values indicates that the use of this oil will be effective at a concentration of 0.9%, which will kill 99.9% of the spores of the pathogen after 24 h of incubation compared to the control variant.

For the Greek oregano essential oil, the analysis of the obtained MFC values after a 24 h incubation showed that a 50% *M. mali* conidia death rate occurs at an essential oil concentration of 0.4% (Table 3).

For the thyme essential oil:

- The MIC values were 1.5%, 0.8% and 1.2% after 15 min, 2 h and 24 h, respectively;
- The MFC values were 3.6%, 2.0% and 2.4% after 15 min, 2 h and 24 h, respectively.

For the thyme oil, the analysis of the obtained MIC values indicates that the use of this oil will be effective at a concentration of 1.2%, which will cause the death of 99.9% of the spores of the fungus after 24 h of incubation.

For the thyme essential oil, the analysis of the obtained MFC values after a 24 h incubation showed that a 50% *M. mali* conidia death rate occurs at an essential oil concentration of 2.4% (Table 3).

To confirm the above-described results on the determination of the MIC and MFC values using flow cytometry, the poisoned substrate method was used. The obtained results indicated that both the Greek oregano oil and thyme oil inhibited the growth of the fungal colony (Table 4).

## 3. Discussion

Due to their high antimicrobial activity, EOs are widely used in the food, cosmetic and pharmaceutical industries. The EOs studied in this paper differed significantly in terms of chemical composition. Depending on the main component, several chemotypes, both pure and mixed, based on the dominant compounds, are distinguished in an EO. In the case of pure chemotypes, the main compound constitutes more than 50% of the total fraction, thus allowing for a clear chemotype determination [20].

*Origanum vulgare* ssp. *hirtum*—Greek oregano—is a typically Mediterranean subspecies, characterized by a very high EO content of 2.30–7.40% [21]. The most common chemotypes are thymol, carvacrol, thymol–carvacrol and carvacrol–thymol, with pure chemotypes being found much more commonly than mixed chemotypes [22]. In the Greek oregano EO obtained by the authors of this paper, the dominant compounds were monoterpenes: phenolic monoterpens, with carvacrol (60.65%) as the dominant component, and monoterpene hydrocarbons, with the domination of γ-terpinene (17.04%). Thus, this EO may be classified as pure carvacrol chemotype. In total, 26 components were identified in the Greek oregano EO compared to the 21 reported by Kosakowska et al. [23] and the 39 by Konakchiev et al. [21].

In the thyme EO, the authors of this paper identified 27 compounds, with a clear dominance of thymol (57.06%), p-cymene (16.56%) and γ-terpinene (7.33%). Thyme is a plant that exhibits a high variability in the chemical composition of its EO. Currently, many chemotypes of thyme EO are known, including geraniol, linalool, 1,8-cineole, thujanol, terpineol, carvacrol and thymol [24]. Despite such a large diversity within thyme, thymol is by far the most common chemotype [20,25,26,27,28]. These data are also confirmed by the results obtained in our work, in which the thyme EO was recognized as a pure thymol chemotype. It comprised of 27 components, while Król and Kiełtyka-Dadasiewicz [28] reported 40 constituents.

The authors of the presented paper identified 24 components of the costmary EO, while Bączek et al. [29] described the presence of 49 compounds. Analysis of the chemical composition of the costmary EO showed that it mainly consisted of compounds classified into the monocyclic monoterpenes group, in which the dominant component was β-thujone (89.38%). Thus, it may be classified as pure β-thujone chemotype. To data, only a few costmary chemotypes have been recognised: carvone, camphor, camphor–tujone and carvone–α-thujone. According to the literature, the most common costmary chemotype is the carvone one; however, information on the presence of the pure β-thujone chemotype can also be found [29,30,31].

Among the known essential oils, there are those that exhibit a high fungistatic activity. The mechanism of their action is probably due to their lipophilic character; therefore, interaction with the fungal cell membranes occurs. As a result of ergosterol binding and the inhibition of cell membrane biosynthesis, the cell membrane is destroyed and the cellular components leak outside. Essential oils contribute to energy loss in cells, which is the result of inhibition of the respiratory chain in mitochondrial membranes, inhibition of proton pumps and a decrease in ATP production [32,33]. In addition, the inhibitory effect of essential oils was observed on the polymerization of chitin, and this had an influence on the maturation of the fungal cell wall, the formation of septa and the disruption of cell division and cell growth [32].

In the literature on the subject, information can be found on the fungistatic effect of various essential oils in terms of the development of various plant-pathogenic fungi [34,35,36,37,38,39,40,41].

Different fungal species may differ in their sensitivity to the effects of the same essential oils. The therapeutic properties of essential oils are very diverse and most commonly related to the effects of the dominant components. However, it should be taken into account that an essential oil, depending on its origin, may be composed of many other compounds that are present at lower concentrations. Thus, the biological activity of essential oils is the result of the activity of the individual dominant components or the synergistic activity of the compound complex, as described above [42,43]. The antimicrobial activity of oils may be influenced not only by differences in the percentage content of the individual components in the oil, even when it is derived from the same plant species, but also, among others, the place of origin, including the soil conditions or climate zone, and the raw material used [44,45,46,47]. The sensitivity of fungi of the genus *Alternaria* to the effects of the thyme oil was stronger compared to the sensitivity of fungi of the genus *Cladosporium* or *Trichoderma* [48]. Fungal species of the genus *Penicillium* were more susceptible to the effects of the oregano oil than the species *Claviceps purpurea* (Fr.) or *Monilinia fructigena* (Pers.) [49]. The results of the studies conducted by Sadowska et al. [50] indicate that among the six tested essential oils—thyme, grapefruit, rosemary, geranium, lemongrass and tea tree—the strongest fungistatic properties against the tested *Sclerotinia sclerotiorum*, *Phoma lingam*, *Rhizoctonia solani* and *Fusarium oxysporum* fungi was exhibited by the thyme oil which, when added to the medium in concentrations of 0.2 and 0.02%, caused a 100% inhibition of the growth of all the tested pathogens. The fungi *Cladosporium cladosporioides*, *Alternaria alternata* and *Chaetomium globosum* exhibited a greater sensitivity to the effects of the oregano oil than the thyme oil. The oregano oil inhibited the development of fungi such as *C. cladosporioides*, *Alternaria alternata* and *Chaetomium globosum*, whereas the thyme oil did not exhibit a fungicidal activity against *C. cladosporioides* [51]. The fungi of the *Aspergillus* genus were also characterized by a varied susceptibility to the effects of the aforementioned essential oils. The fungistatic effect of the oregano oil against the fungi of the genus *Aspergillus* was stronger compared to the fungistatic effect of the thyme oil [52,53]. Daferera et al. [54] noted that the fungistatic activity of the oregano oil against the fungi of the genus *Fusarium* and the species *Botrytis cinerea* (Pers.) was more efficient compared to the activity of the thyme oil. Wójcik-Stopczyńska and Jakubowska [55] noted that adding thyme, basil and rosemary essential oils to the medium, instead of the oregano oil, resulted in a more efficient inhibition of the development of the fungi *Alternaria alternata*, *Aspergillus flavus*, *A. fumigatus*, *A. niger*, *Cladosporium herbarum*, *Fusarium oxysporum*, *Penicillium cyclopium*, *Eurotium amstelodami*, *E. chevalieri*, *E. herbariorum*, *E. repens* and *E. rubrum*. The results of the study conducted by Sampietro et al. [56] indicate the fungistatic effect of β-thujone, the dominant component of the costmary oil, on fungi of the genera *Aspergillus*, *Septoria* and *Fusarium*.

The set of essential oils, their concentrations and their incubation times selected for the experiments presented by the authors of this paper were determined on the basis of the data on the influence of essential oils, i.e., the thyme and oregano essential oils on the development of yeast of the genus *Zygosaccharomyces* [57], the bacteria *Escherichia coli*, *Klebsiella oxytoca* and *Klebsiella pneumoniae* [58] and the fungi *Monilinia fructigena* and *Penicillium expansum* [49]. The results of the studies, as described by the indicated authors, indicated a markedly stronger fungistatic activity for the oregano oil compared to the thyme oil. Similarly, the results presented in this paper indicate that among the three essential oils (Greek oregano oil, thyme oil and tansy oil) selected for the study, the Greek oregano oil had the strongest fungistatic activity. The mortality of the *M. mali* conidia was more than 1.5 and 5 times higher when using the Greek oregano oil compared to when using the thyme and costmary oils, respectively, at the same oil concentrations and exposure times.

The differences in the fungistatic activity of the tested essential oils against the *M. mali* fungus may result from differences in the structure of their dominant components and the diversity of their chemical composition, thus the presence and concentration of the active components [59].

The above-performed analysis of the composition of the essential oils studied in this paper shows that the dominant components of the Greek oregano oil were carvacrol and γ-terpinene.

In the thyme oil, thymol, p-cymene and γ-terpinene were present at the highest amounts. These compounds are biosynthetically related. When γ terpinene is aromatised, p-cymene is formed, which is then converted by hydroxylation to thymol and its isomer carvacrol. Both γ-terpinene and p-cymene (monocyclic monoterpenes), as well as thymol and carvacrol (phenolic monoterpenes), exhibit a strong fungistatic activity. This fungistatic activity is markedly stronger for thymol and carvacrol than for γ-terpinene and p-cymene [60]. These substances constituted the dominant compounds in the oregano oil, with carvacrol being present in the largest amount.

The sensitivity of *M. mali* to the effects of the essential oils depended not only on the concentration of the oil used in the medium but also on the time that elapsed after its use. The strongest fungistatic properties against the tested fungus were exhibited by the Greek oregano oil at a concentration of 0.9%, the addition of which to the medium resulted in a 56.17%, 69.68% and 96.05% inhibition of growth of the fungus after 15 min, 2 h and 24 h, respectively. In the remaining combinations, the inhibition of fungal growth was significantly weaker.

The antifungal activity of essential oils is determined by establishing the minimum concentration of the compound causing a 90% inhibition of growth of the fungal cells, i.e., the so-called MIC, and the minimum fungicidal concentration (MFC) of the tested active substance needed to kill 99.9% of the initial inoculum after incubation for 24 h under standardized conditions [61].

Frankova et al. [49], when testing the fungicidal activity of four essential oils (cinnamon, lemongrass, clove and oregano), found that the latter, the oregano essential oil, was distinguished by the most efficient activity against a complex of post-harvest apple pathogens, including *Penicillium expansum*. The MIC value for this oil ranged from 0.0032 to 0.0128%.

Similarly, in the experiments described by Puškárová et al. [51], the oregano oil exhibited an efficient inhibitory activity against five fungal species (*Ch. globosum*, *C. cladosporioides*, *A. alternata*, *Aspergillus fumigatus* Fresen. and *Penicillium chrysogenum* Thom). The MIC value for this oil was in the range of 0.01–0.025%, and the MFC value ranged from 0.025 to 0.075%.

In the case of fungicidal activity, the thyme oil showed a weaker activity than the oregano oil. When tested against the species susceptible to the thyme oil belonging to the genera *Ulocladium* and *Alternaria*, the MIC value was 0.545–0.985%. In the case of fungi less sensitive to the activity of the thyme oil (e.g., *Cladosporium* spp., *Trichoderma* spp. and *Penicillium* spp.), the MIC value ranged from 1.5 to 1.9% [48].

Although the microdilution method remains a common, fundamental and standardized approach for antimicrobial susceptibility testing (AST), flow cytometry presents an intriguing alternative. Flow cytometry is a highly sensitive technique that enables multiparametric analysis of cells based on their morphological and phenotypic characteristics. One of its key advantages is the ability to assess specific cellular responses at the single-cell level, providing detailed insights into population heterogeneity and allowing visualization of parameter distribution across the tested sample. Flow cytometry fulfils the essential criteria for an effective AST method: it is rapid, reliable, straightforward and broadly applicable to various microorganisms. Notably, it allows for analysis using minimal sample volumes, including low concentrations of test compounds, and can deliver results within a single day. These features make it a promising alternative, particularly for preliminary assessments and high-throughput screening applications.

Flow cytometry can be utilised in studies on the antimicrobial activity of many substances, including vegetable essential oils. Tian et al. [62] determined the fungistatic activity of fennel essential oil against *Aspergillus flavus* by using propidium iodide staining to demonstrate cytoplasmic membrane damage. Green et al. [63] described the use of a flow cytometer to determine the MIC and MFC values in studies on the mortality of the *Candida albicans* yeast. They described the method as fast, reliable and adaptable, and they considered the device itself as useful for these types of studies. Six years later, Ramani and Chaturvedi [64] conducted research on the usefulness of flow cytometry in a study on the antifungal activity of Amphotericin B against various fungal species within the *Candida* genus: *C. glabrata*, *C. guilliermondii*, *C. krusei*, *C. lusitaniae*, *C. parapsilosis*, *C. tropicalis* and *Cryptococcus neoformans*. The researchers also described this method as simple and potentially useful for conducting this type of research. Subsequently, Pina-Vaz et al. [65] used a flow cytometer to study the influence of thyme oil on the development of fungi of the genus *Candida*. The researchers emphasized the usefulness of this method in studying the antifungal properties of the oil in short incubation times. A considerable part of the available literature refers to the usefulness of flow cytometry in studying the effects of various substances on single-celled organisms, i.e., yeasts. Studies on simple organisms, divided by a maximum of one septum (in the case of spores), are much easier to optimise than studies on multi-celled organisms separated by more than one septum. This is due to the fact that the device gives the result for individual cells and, consequently, in the case of multiple septa in one spore, the cytometer treats them as separate, unconnected cells. Therefore, in the case of fungi such as *M. mali*, where microcyclicity occurs and leads to the formation of conidia separated by multiple septa, it is very important to delay this process to the maximum degree. In the conducted studies, the authors of this paper used a flow cytometer to obtain the MIC values, which ranged from 0.8 to 0.4, and the MFC values, which range from 1.6 to 0.9, for the Greek oregano oil. For the thyme oil, the MIC values ranged from 1.5 to 1.2 and the MFC values from 3.6 to 2.4.

In the literature on the subject, there is no data on the fungistatic activity of essential oils against the *M. mali* fungus. The results of the experiments presented in this paper are innovative and are the first of this type published in the world literature on the fungistatic activity of the essential oils extracted from Greek oregano, thyme and costmary against *M. mali*, as determined using a flow cytometer. The issues addressed in this paper constitute a very important first step in studies on the use of essential oils to protect apple trees from the sooty blotch and flyspeck disease and require further research.

The search for new, efficient and, most importantly, environmentally safe methods of crop protection has become a priority task. The experiments described in this paper provide new information on the use of alternative products, i.e., natural plant-derived products with antimicrobial properties, for the protection of plants against fungi. These criteria are met perfectly by essential oils, which are products of secondary plant metabolism and exhibit a number of valuable biological properties, including antibacterial activity. The attractiveness of essential oils as compounds with fungistatic activity is all the more interesting because, due to their rich composition, the mechanisms by which fungi acquire resistance to these metabolites have not been found. In the future, these natural products may be applied to protect apple trees from sooty blotch and flyspeck in both conventional, integrated and organic cultivation systems.

## 4. Materials and Methods

### 4.1. Plant Raw Materials Used for EO Distillation

The EOs used in the study were obtained from the herbs of three species of medicinal and aromatic plants—Greek oregano (*Origanum vulgare* L. subsp. *hirtum* (Link) Ietswaart), thyme (*Thymus vulgaris* L.) and costmary (*Tanacetum balsamita* L.). The plants—Greek oregano, thyme and costmary—were cultivated at the experimental field of the Department of Vegetable and Medicinal Plants (Institute of Horticulture Sciences, Warsaw University of Life Sciences-SGGW).

### 4.2. EO Extraction and GC-MS/GC-FID Analysis

The essential oils were obtained according to the European Pharmacopoeia, with modifications [66]. An amount of 50 g of air-dried raw material was used for hydrodistillation (3 h) in a Deryng-type apparatus. Until further analysis, the samples were stored in dark vials at 4 °C.

Analysis of the essential oils was conducted using gas chromatography–mass spectrometry (GC–MS) with a flame ionization detector (FID). The qualitative and quantitative analysis was carried out by using an Agilent Technologies 7890A gas chromatograph equipped with an FID and MS Agilent Technologies 5975C Inert XL_MSD with Triple Axis Detector (Agilent Technologies, Wilmington, DE, USA). The details of the working conditions were reported previously by Bączek et al. [67]. Polar, capillary column HP 20 M (25 m × 0.32 mm × 0.30 µm) (Agilent Technologies, Wilmington, DE, USA) was applied. The oven temperature started from a level of 60 °C for 2 min, with the temperature rising at a rate of 4 °C/min from 60 °C to 220 °C, then held isothermally at 220 °C for 5 min. The carrier gas (He) flow rate was 1.1 mL/min. The split ratio was 1:50. A total of 1 µL of the diluted samples (1/100 *v*/*v*, in n-hexane/isopropanol) was injected at 210 °C by an auto sampler. The ion source temperature was −220 °C, the ionization voltage was 70 eV and the range of mass spectra scanning was 40–500 amu. EO compound identification was based on mass spectra from the databases NIST08, NIST27, NIST147, NIST11 and Wiley7N2 and on comparison of retention indices (RIs) relative to the retention times of a series of n-alkanes (C7–C30) (Merck KGaA, Darmstadt, Germany) with those given in the literature [19]. The proportions of the compounds identified in the EOs were computed by the normalization method from the GC peak areas.

### 4.3. Influence of the Concentrations of the Essential Oils on the Viability of the M. mali Conidia

The studies were conducted using a BD FACSVerse™ flow cytometer (San Jose, CA, USA) and a BD FACSuite™ computer program coupled thereto. For the studies, the CISZYj3p20 isolate of the fungus *M. mali*, the causal agent of sooty blotch and flyspeck (a collection of the Department of Plant Protection, Institute of Horticultural Sciences, Warsaw University of Life Sciences-SGGW—WULS-SGGW), was selected; it had been isolated from apples originating from the town of Ciszyca near Warsaw (Masovian Voivodeship) in 2008. This isolate sporulated abundantly, formed secondary conidia belatedly by germination of the spores already produced (microcyclic conidiation) and formed mycelial hyphae on agar media relatively belatedly, which was very important for the studies carried out with the cytometer.

Analysis of the viability of the *M. mali* conidia involved fluorescein diacetate (FDA). The FDA assay is a widely used method for quantifying viable cells by assessing their metabolic activity. It is commonly applied across various fields—including microbiology, medicine, and ecology—to study different cell types and to gain insights into cell population characteristics at the single-cell level. FDA is a nonpolar, hydrophobic compound that can readily diffuse across cell membranes. Once inside living cells, it is hydrolysed by nonspecific esterases, producing fluorescein, a bright green fluorescent xanthene derivative. The primary enzymes responsible for this hydrolysis are typically lipases and acylases. For the purpose of the FDA assay, a 5 mg/mL stock solution of FDA (SSFDA) was prepared in acetone. The resulting solution was filtered and stored in dark glass vials at –20 °C. The working solution was freshly prepared immediately before staining by diluting the SSFDA stock 20-fold with distilled water.

The *M. mali* conidia were transferred with a sterile needle into sterile plastic Petri dishes (dia. 8 cm) filled with an agar medium—WA (Water Agar) [68]—and incubated for 24 h at room temperature in daylight. Subsequently, the colonies of the fungus were transferred with a sterile, plastic inoculation loop to 30 mL of deionized water contained in a sterile conical flask with a capacity of 50 mL. The output solution prepared in this manner was filtered with a CellTrics^®^ filter (Sysmex Partec GmbH, Orifiz, Germany) with a pore diameter of 50 μm. Subsequently, 0.75 mL of this solution was added to Eppendorf-type tubes (1.5 mL) and made up to 1 mL with deionized water. The control variant of the experiment was constituted by a diluted stock solution without the addition of an essential oil. The following concentrations of the oils were used in the tests: 0.09, 0.1, 0.2, 0.3, 0.4, 0.5, 0.6, 0.7, 0.8 and 0.9%. The samples were incubated for 30 min at room temperature, then 10 µL of a fluorescenine diacetate solution (FDA) was added to the tubes and each of the samples was incubated in total darkness (room temperature) for 15 min, 2 h and 24 h in order to compare the influence of time on the effects of the essential oils. Before the measurements were carried out, the samples were each transferred to a refrigerator (4 °C) in order to inhibit the enzymatic processes for the duration of the measurements. In each sample, the cytometric analysis covered up to 10,000 spores. The experiment on the viability of the *M. mali* conidia for all EO treatments was replicated twice. Two biological repetitions were applied for each of the EO concentrations.

Photographic documentation of the fluorescence of the conidia was carried out with a BX50 light microscope (Olympus, Tokyo, Japan), a DP71 camera (Olympus) cooperating therewith, and a Cell-F computer program (Olympus). To induce the fluorescence of the spores, a mercury fluorescent lamp (Olympus) compatible with the microscope was used.

### 4.4. Statistical Analysis of the Obtained Results

Statistical analyses of the obtained results of the experiments were carried out with the RStudio 2024.04.2 program.

In order to determine the influence of the applied concentrations of the different essential oils (from Greek oregano, thyme and costmary) and the conidia incubation time on the viability of the *M. mali* conidia, a three-way analysis of variance (MANOVA) was performed. For comparisons of the effects of the individual factors, a Newman–Keuls multiple comparisons test was used at a significance level of *p* = 0.05.

The relationships between the concentration of the applied essential oils and the viability of the *M. mali* conidia were determined by performing a linear regression analysis.

y = a + bx

where y is spore viability (in %), x is essential oil concentration, a is the intercept (constant) and b is the slope coefficient.

Regression equations were employed to calculate the minimum inhibitory concentration (MIC) and minimum fungicidal concentration (MFC). The lowest concentration of an essential oil causing a 50% inhibition of the fluorescence of the *M. mali* conidia was taken to be the MIC value. The lowest concentration of an essential oil having a 99.9% inhibition of the fluorescence was taken to be the MFC value.

### 4.5. Influence of the Concentrations of the Selected Essential Oils on the Development of the M. mali Colonies in In Vitro Tests

To confirm the results of the flow cytometry studies on the influence of the concentration of the selected essential oils on the viability of the *M. mali* conidia, a poisoned substrate method, as described by Kowalik and Krechniak [69], was utilised.

PDA medium (1000 mL) was poured into round-bottom flasks with a capacity of 250 mL and an appropriate amount of an essential oil was added to each of them to obtain the concentrations of the essential oils, which were 1.2 and 2.4% for the thyme oil and 0.4 and 0.9% for the Greek oregano essential oil. For each of the selected concentrations of the essential oils, 10 sterile Petri dishes (dia. 30 mm) were prepared. The experiment was performed in triplicate. The control variant comprised 10 Petri dishes filled with a PDA medium without the addition of the aforementioned essential oils. The inoculum consisted of medium discs overgrown with *M. mali* mycelium that had been cut from 14-day-old cultures with a sterile corkborer (dia. 5 mm) and transferred with a preparation needle to a PDA medium with the addition of an essential oil.

The fungus-inoculated medium in the Petri dishes was incubated at a temperature of 17 °C and illuminated with fluorescent lamps (14 h day/10 h night). Measurement of the size of the *M. mali* colony was carried out after 14 days. The diameter of the fungal colony was measured using a ruler. The percentage of colony growth inhibition was calculated according to the formula

$$\%\;growth\;inhibition=\frac{\left(Ko-F\right)}{Ko}\cdot 100$$

where *Ko* is the culture diameter in the control variant and *F* is the culture diameter in combination with an essential oil

## 5. Conclusions

Based on the conducted experiments, both Greek oregano EO and thyme EO demonstrated fungistatic activity against the *M. mali* fungus.

The carvacrol-rich essential oil (the Greek oregano EO) caused higher mortality in the *M. mali* conidia compared to the thymol- and β-thujone-rich essential oils (the thyme EO and costmary EO, respectively).

Flow cytometry finds use as a method to study the fungistatic effects of essential oils on fungi producing homogeneous spores.

The results of the research presented by the authors of this paper are the first step towards beginning work on the utilisation of the fungistatic activity of essential oils, especially the oregano oil, for the protection of apple trees against the *M. mali* fungus, the causal agent of sooty blotch and flyspeck.

## Figures and Tables

**Table 1:** Composition of the EO samples.

				Relative Area (%)		
No.	Compound	RI ^1^	RI ^2^	Greek Oregano	Thyme	Costmary
1	(Z)-salvene	935	-	-	-	0.21
2	(E)-salvene	949	-	-	-	0.07
3	α-thujene	1023	1012–1039	0.17	0.35	0.03
4	α-pinene	1029	1008–1039	1.54	1.72	0.05
5	camphene	1075	1043–1086	0.41	0.25	0.23
6	β-pinene	1111	1085–1138	0.67	0.19	0.14
7	sabinene	1125	1098–1140	0.03	0.08	0.04
8	β-myrcene	1167	1155–1169	2.47	1.06	-
9	α-terpinene	1183	1154–1195	2.17	1.38	0.34
10	limonene	1203	1178–1219	0.29	0.25	0.36
11	1.8-cineole	1216	1186–1231	-	0.04	2.43
12	β-ocimene	1236	1211–1251	0.01	0.07	-
13	γ-terpinene	1253	1222–1266	17.04	7.33	0.11
14	*p*-cymene	1276	1246–1291	5.43	16.56	0.58
15	terpinolene	1285	1261–1300	0.05	0.07	0.11
16	hexanol	1349	1344–1360	-	0.14	-
17	octan-3-ol	1392	1372–1408	-	0.24	-
18	α-thujone	1418	1385–1441	-	-	2.81
19	β-thujone	1437	1400–1452	-	-	89.38
20	1-octen-3-ol	1446	1411–1465	0.54	-	-
21	menthone	1459	1450–1475	0.40	-	0.06
22	α-copaene	1494	1462–1522	-	0.71	-
23	camphor	1509	1481–1537	0.19	0.24	0.10
24	β-cubebene	1536	1518–1560	-	1.49	-
25	linalool	1542	1507–1564	0.47	1.6	0.05
26	bornyl acetate	1577	1549–1597	0.94	0.96	-
27	β-caryophyllene	1593	1570–1685	-	0.51	0.20
28	terpinen-4-ol	1597	1564–1630	0.59	0.65	-
29	pulegone	1623	1626–1663	-	-	0.29
30	menthol	1631	1599–1651	0.17	-	0.11
31	α-terpineol	1680	1682–1706	1.02	-	-
32	borneol	1687	1653–1728	-	0.23	-
33	carvone	1711	1699–1751	0.42	-	-
34	germacrene D	1716	1676–1726	0.32	0.14	-
35	caryophyllene oxide	1976	1936–2023	-	0.44	-
36	(−)spathulenol	2124	2074–2150	0.12	-	-
37	thymol	2164	2100–2205	0.98	57.06	0.25
38	α-bisabolol	2197	2178–2234	0.19	-	-
39	carvacrol	2211	2140–2246	60.65	3.59	0.26
40	β-eudesmol	2235	2196–2272	-	-	0.15
	Total identified			97.28	97.35	98.36
	Monoterpene hydrocarbons			30.28	29.31	1.99
	Oxygenated monoterpenes			4.20	3.72	95.23
	Phenolic monoterpenes			61.63	60.65	0.51
	Sesquiterpene hydrocarbons			0.32	2.85	0.20
	Oxygenated sesquiterpenes			0.31	0.44	0.15
	Other compounds			0.54	0.38	0.28

RI ^1^—experimental retention index on polar column; RI ^2^—range of retention indices on polar column reported by Babushok et al. [19].

**Table 2:** Influence of the essential oils on the viability of the *Microcyclosporella mali* conidia.

Essential Oil	Average Viability of Conidia (%)
Costmary	91.96 a *
Thyme	74.31 b
Greek oregano	57.13 c

* homogeneous groups according to the Newman–Keuls test. Values marked with the same letter are not significantly different at the significance level of *p* = 0.05.

**Table 3:** The minimum inhibitory concentration (MIC) and minimum fungicidal concentration (MFC) values for the Greek oregano and thyme essential oils after 15 min, 2 h and 24 h of incubation of conidia.

Essential Oil	Incubation Time	MIC	MFC
Greek oregano	15 min	0.8	1.6
	2 h	0.5	1.3
	24 h	0.4	0.9
Thyme	15 min	1.5	3.6
	2 h	0.8	2.0
	24 h	1.2	2.4

**Table 4:** Inhibition of the growth of the *Microcyclosporella mali* fungal colony after using different concentrations of the essential oils.

Essential Oil	Concentration (%)	Inhibition of the Growth of the Colonies (%)
Greek oregano	0.4	62
	0.9	65
Thyme	1.2	60
	2.4	64

## Data Availability

No new data were created or analyzed in this study. Data sharing is not applicable to this article.

## Supplementary Materials

The following supporting information can be downloaded at: https://www.mdpi.com/article/10.3390/molecules30153122/s1, Table S1: The results of the multi-way analysis of variance concerning the influence of 3 factors: an essential oil, the incubation time, and the concentration of the essential oil, on the viability of the *Microcyclosporella mali* conidia. Table S2: Regression equations for essential oils describing viability of *Microcyclosporella mali* spores after 15 min, 2 h and 24 h.
