# Supplementary material for: A Preliminary Study of the Response of Microcyclosporella mali to Selected Essential Oils
Source: Molecules. 2025 Jul 25;30(15):3122. doi: 10.3390/molecules30153122 (PMC12348070; doi:10.3390/molecules30153122)
Supplement: Supplementary file 1 [file molecules-30-03122-s001.zip › molecules-3730226-supplementary.pdf]

Table S1: The results of the multi-way analysis of variance concerning the influence of 3 factors: an essential oil, the incubation time, and the concentration of the essential oil, on the viability of the *Microcyclosporella mali* conidia

Source of variance	Sum of squares	Degrees of freedom	Variance	F	p
**Effect of the factors**					
**A:** essential oil	54587.5	2	27293.7	605.56	0.0000
**B:** incubation time	5774.48	2	2887.29	64.06	0.0000
**C:** concentration of the essential oil	34153.4	9	3794.82	84.19	0.0000
**Interaction of the factors**					
AB	8187.0	4	2046.75	45.41	0.0000
AC	25822.1	18	1434.56	31.83	0.0000
BC	3689.39	18	204.966	4.55	0.0000
Residual (random error)	9735.56	216	45.072		

Tables S2: Regression equations for essential oils describing viability of *Microcyclosporella mali* spores after 15 min, 2 h and 24 h

Esential oil	After	Regression equation	R^2
Greek oregano	15 min	Y*= 96.55 – 59.41x C**	77.82
Greek oregano	2 h	Y=89.97 – 73.67 x C	75.66
Greek oregano	24 h	Y= 95.48 – 104.92 x C	88.48
Thyme	15 min	Y=90.40 – 29.96 x C	57.83
Thyme	2 h	Y=88.43 – 49.18 x C	74.25
Thyme	24 h	Y=101.337 – 41.80 x C	54.72

* Viability (%)

**Essential oil concentration
